# Chemical and Microstructural Properties of Designed Cohesive M-S-H Pastes

**DOI:** 10.3390/ma15020547

**Published:** 2022-01-12

**Authors:** Charlotte Dewitte, Alexandra Bertron, Mejdi Neji, Laurie Lacarrière, Alexandre Dauzères

**Affiliations:** 1 Institut de Radioprotection et de Sûreté Nucléaire (IRSN), PSE-ENV/SEDRE/LETIS, 92260 Fontenay-aux-Roses, France; charlotte.dewitte@irsn.fr (C.D.); mejdi.neji@irsn.fr (M.N.); alexandre.dauzeres@irsn.fr (A.D.); 2 LMDC (Laboratoire Matériaux et Durabilité des Constructions), Université de Toulouse, UPS, INSA, 135 Avenue de Rangueil, CEDEX 04, 31077 Toulouse, France; laurie.lacarriere@insa-toulouse.fr

**Keywords:** magnesium silicate hydrates (M-S-H), X-ray diffraction, thermogravimetric analysis, autoradiography, physisorption, mercury porosity

## Abstract

Concretes can be exposed to a magnesium attack in several environments leading to the formation of magnesium silicate hydrates (M-S-H) and brucite (MH). The formation of M-S-H is likely to alter the properties of the cement matrix because it is linked to the decalcification of C-S-H. However, relatively few data on M-S-H exist in the literature. In order to characterize, physically and mechanically, the M-S-H phase, pure M-S-H cohesive pastes are needed. This work studies the formation of cohesive M-S-H pastes made with MgO-to-SiO_2_ atomic ratios of 0.78, 1 and 1.3, from two types of silica (silica fume or colloidal silica) and under 20 °C and 50 °C thermal curing. X-ray diffraction and thermogravimetric analyses confirmed that the consumption of brucite and the formation of M-S-H were quicker with a 50 °C curing. Energy-dispersive X-ray spectroscopy and microtomography showed that colloidal silica enabled a better distribution of the particles than silica fume. Microstructural characterizations were conducted under the protocol with colloidal silica and 50 °C thermal curing. Porosity investigations allowed to describe the M-S-H pastes as highly porous materials with a low content of micropores in comparison with mesopores. The type of mixing influenced the mesopore size distribution.

## 1. Introduction

Magnesium (Mg) enrichment of cementitious materials has been observed in civil engineering construction exposed to three types of aqueous environments: seawater, soft water (in contact with marine construction and dams [1,2,3,4,5]), and pore water of clayey rock (in contact with concrete intended for disposal galleries or bentonite plug support blocks in deep radioactive waste repositories [6,7,8,9,10,11,12]). Real-life aqueous environments are often multi-component, generating a combination of chemical attacks on concrete. In seawater, Mg attacks act in conjunction with carbonate, chloride and sulphate ions [2,4,13,14]. In groundwater environments and/or geological environments intended for radioactive waste repository, Mg enrichment—associated with decalcification, carbonation and sulphate attack—has been reported at the interface between cement-based materials and clayey rock [6,7,8,9,10,11,12]. The same perturbation has been observed during contact between soft water and dams [5]. The degradation can be divided into several physical zones where the action of sulphates, decalcification and carbonation combine with Mg enrichment.

During Mg attacks, magnesium reacts mainly with the calcium phases of the cement matrix—portlandite (Ca(OH)_2_ or CH in cement notation) and calcium silicate hydrates (C-S-H) in particular—to form brucite and magnesium silicate hydrates (M-S-H). For ordinary binders with a high calcium-to-silicon ratio (Ca/Si), i.e., Portland cements, Mg attacks have been evidenced by the formation of brucite from the dissolution of portlandite. Brucite precipitates first at the surface of concrete specimens, reducing the kinetics of further species diffusion. In the long term, a slow decalcification of C-S-H leads to the formation of M-S-H [2,6,9,15].

For binders with a low Ca/Si ratio, the Mg attack mechanisms are different. These binders have been developed for the following purposes: (i) to reduce heat release leading to early-age cracking risks for massive structures [16]; (ii) to improve the long-term mechanical performance, durability and sustainability of concrete (to resist penetration of chloride ions, for example) [17,18]; and (iii) to reduce environmental impact [19]. Part of the clinker is substituted by several materials such as fly ash, silica fume or slag. The consequences of these substitutions are multiple: improvement of the granular skeleton; reduction of heat release by hydration reactions; reduction of pH of the interstitial solution [20]; reduction of the Ca/Si ratio of hydrated products [21], etc. These low-heat or low-pH binders are taken into account for the construction of dams [22,23] or the construction of a deep disposal for radioactive waste [7,24], seeking to preserve the clayey rock from the alkaline plume and/or to decrease the thermal effect during concrete hardening. The pH of the matrices obtained by hydration of these binders is low (11 < pH < 12.5) [25] and the pastes obtained do not contain much—or any—portlandite. Mg attacks on such materials are characterized by a fast decalcification of C-S-H and the formation of M-S-H [6].

When hydrated cement pastes (containing portlandite, aluminates and C-S-H) are subjected to pure water, decalcification has deleterious effects on the physical and mechanical properties of concrete, mainly by increasing porosity and decreasing mechanical performance [26,27,28,29,30,31]. In the case of magnesium-rich water, the positive or negative effect of M-S-H formation on the mechanical and physical properties degraded by decalcification is not known.

In degraded cement pastes or concrete, M-S-H were found to be intermixed with other phases, thus complicating the identification of their intrinsic properties [5,11,12,24]. Synthetic M-S-H powders (produced from MgO, SiO_2_ and water) have already been developed to analyze the formation of the phase (in terms of formation kinetics and equilibrium constants), its chemical and mineralogical characteristics, and the structure of the M-S-H gel [32,33,34,35,36,37,38,39,40,41,42,43]. When water, MgO and SiO_2_ react, the M-S-H are not the phase preferably formed at an early stage. The main precipitated phase is brucite (Mg(OH)_2_) due to the slower dissolution of silica compared to magnesium [37,44]. For reactive transport modeling (Hytec, for instance), it is necessary to know the physical properties of M-S-H to understand how the porosity evolves in decalcified materials when M-S-H precipitate. In order to characterize the microstructure of M-S-H gels, cohesive M-S-H pastes are needed.

The preparation of pure cohesive M-S-H pastes faces two main obstacles: (i) the difficulty to obtain cohesive pastes with representative porosity and microstructure that allow the physical and mechanical characterization of in situ, experimentally observed M-S-H; and (ii) the difficulty to obtain materials containing only M-S-H phases within a reasonable time (a few months).

Concerning the first aspect, two protocols for manufacturing M-S-H pastes have been developed in the literature to meet the high need of water of MgO powder [45], either using a high water/binder ratio (w/b = 2) [44,46] or sodium hexametaphosphate (NaHMP), a superplasticizer (0.025–0.3 g in 10 g of water, with 0.4 < w/b < 0.8) [45,47]. The influence of NaHMP on the structure of M-S-H and other hydrated phases has been studied by several authors [48,49,50]. Although it led to a better rheological behavior and the formation of M-S-H over brucite (due to the adsorption of phosphate species on MgO, which inhibits the nucleation of the Mg(OH)_2_), the formation of secondary phases (such as $\left[6{\mathrm{MgOH}}^{+}\cdot {\left({\mathrm{PO}}_{3}\right)}_{6}^{6-}\right])\;$[49,50] and minor changes in the structure of M-S-H gels have been observed [48,50]. NaHMP could thus cause chemical and mineralogical differences between in situ precipitated M-S-H and synthetized M-S-H. At high w/b ratios, the structure could be modified. Excess of water results in the modification of the porous network through the creation of pores, air voids, etc., which alters species diffusion. Moreover, high porosity weakens the material and complicates mechanical characterization.

Concerning the second aspect, literature on M-S-H powder synthesis has shown that the mechanism of M-S-H formation from the reaction between silica fume and MgO is a slow process that lasts at least 2–3 years at 20 °C [34,43]. Several researchers [36,39,45,46] have reported that M-S-H are not the only phase formed during the initial contact between MgO, silica fume and water at 20 °C. Silica fume dissolves more slowly than MgO reacts, leading to thermodynamic conditions favorable to brucite formation (low silicon concentration in the solution). Brucite is subsequently consumed, reacting with the amorphous silica to form M-S-H. This process leads to a very long synthesis. This problem can be circumvented by increasing the curing temperature as shown by Bernard [36], who obtained M-S-H powders cured at 50 °C or 70 °C comparable to those cured at 20 °C but with a shorter hydration time. Bernard showed that a cure at 50 °C or 70 °C on M-S-H powders seemed to reduce the content of brucite and improved the reaction to the advantage of M-S-H. The effect appeared to be the same at 50 °C and 70 °C. Another option to quickly obtain pastes containing only M-S-H could be to use a finer silica, e.g., colloidal silica.

This paper aims to develop a protocol for producing cohesive pastes of M-S-H with high purity and different magnesium to silicon (M/S) ratios, and to microstructurally characterize them to further the knowledge on the intrinsic properties of M-S-H. Four protocols for the production of cohesive M-S-H pastes (with different M/S ratios) are proposed and compared. The two parameters modified throughout the protocols were the source of silica (silica fume and colloidal silica) and the curing temperature (20 °C and 50 °C). Moreover, two types of mixing—mechanical and manual—were tested. The obtained M-S-H pastes were then characterized by X-ray diffraction (XRD), thermogravimetric analysis (TGA), microtomography, and energy-dispersive X-ray spectroscopy (EDS). The morphology of the M-S-H pastes was observed with a scanning electron microscope (SEM). Microstructure and porosity characterizations were performed by autoradiography, mercury intrusion porosimetry (MIP), water saturation and helium pycnometry. The characteristics of the pastes obtained were compared with those reported in the literature.

## 2. Materials and Methods

### 2.1. Raw Materials

M-S-H pastes were produced by mixing a source of silica (silica fume or colloidal silica), magnesium (MgO, to be precise) and distilled water (Milli-Q^®^ water). The silica fume (CONDENSIL S95 DM), composed of 95% SiO_2_ and with a specific surface of 22 m^2^/g, was provided by Condensil^®^. The silica fume was sieved at 315 μm prior to its use in manufacturing M-S-H. The colloidal silica (Rheomac AS 150, also called Mastermatrix 150) was provided by BASF^®^ as an aqueous suspension (dry extract = 52 ± 2.5%).

The magnesium was MgO provided by VWR^®^ (VWR International S.A.S, 1 rue d’Aurion, Rosny-sous-Bois, France), with a content of MgO (on the calcined product) > 97% and a loss on ignition of 8%.

The superplasticizer (CHRYSO^®^Fluid Optima 175) was provided by Chryso France^®^ (ZI du Sauvoy, 5 Rue de la Bizière, 77165 Saint-Soupplets, France).

### 2.2. Preparation of M-S-H Samples

The M-S-H pastes were prepared according to a protocol adapted from Tonelli et al. [46]. In Tonelli’s study, silica fume and MgO were mixed at 20 °C with an MgO/SiO_2_ (M/S) atomic ratio of 1 and a w/b ratio of 2. In the present study, the optimal w/b ratio (i.e., the lowest value to maintain sufficient workability without using a superplasticizer) was sought to favor a more cohesive paste, without segregation and with lower porosity. We carried out two thermal curing protocols to try to maximize the formation of M-S-H in the pastes. The source of silica was varied to accelerate the reactivity and improve the homogeneity of the mix (increasing the specific surface area of the reagents). Three different M/S were selected following the work of Bernard et al. [51]: 0.78, 1 and 1.3, as detailed in Table 1. The first campaign consisted of mixes of MgO, silica fume and Milli-Q water with a w/b of 1. For the second campaign, silica fume was replaced by colloidal silica and superplasticizer was used to improve the workability of the mix. Two mixing methods—mechanical (noted E; rotation speed 140–285 rd/min for 10 min, 450 W) and manual (noted M; 5 min)—were tested. Analysis of the chemical composition and microstructure of M-S-H pastes with different mixing techniques, w/b ratios and therefore porosities provided a wider range of data likely comparable to M-S-H formed by precipitation in cementitious materials. All pastes with silica fume were mechanically mixed. For pastes with colloidal silica, both types of mixing were tested for every M/S. The w/b ratio and the superplasticizer content were modified to improve the workability of the mix for manually mixed pastes (w/b = 1.45).

Two curing protocols were carried out for each type of silica and M/S ratio. Half of the samples were put in a sealed box with water (to keep relative humidity close to 100%, no water bath) and placed in a climatic chamber at 50 °C. The other half was put in a box with water and left at ambient temperature.

An abbreviated notation has been implemented to identify M-S-H pastes with respect to their manufacturing protocol. It begins with “MS” followed by the M/S ratio, the type of silica used (SF for silica fume and CS for colloidal silica), the temperature of curing, and finally the type of mixing (for the pastes with colloidal silica; E for mechanically mixed and M for manually mixed). For instance, MS_0-78_CS_T20_M matches the samples made with an M/S ratio of 0.78, colloidal silica, cured at ambient temperature (20 ± 2 °C) and mixed manually (Table 1).

### 2.3. Chemical, Mineralogical and Microstructural Analysis of Pastes

#### 2.3.1. Mineralogical and Chemical Analyses

X-ray diffraction analyses were performed on powders obtained by crushing the samples, either on a Malvern Panalytical Empyrean instrument, a PANalytical X’Pert Pro operating at 40 kV and 40 mA (Sorbonne Université, Jussieu, Paris, France) with a Cu anti-cathode (λ~1.54 Å), a D8 Advance Bruker operating at 40 kV and 40 mA (LMDC, Toulouse, France) with a Cu anti-cathode (λ~1.54 Å), or a PANalytical Aeris (IRSN, Fontenay-aux-Roses, France) operating at 600 W, 40 kV and 15 mA, with a Cu anti-cathode (λ~1.54 Å). The scanning region was in the range 2Θ = 5°–70° with either: (i) a step size of 0.0131, for a total duration of 4 h (Jussieu, Paris, France); (ii) a step size of 0.020, for a total duration of 2 h (LMDC, Toulouse); or (iii) a step size of 0.0109, for a total duration of 20 min (IRSN, Fontenay-aux-Roses, France). X-ray diffractograms were plotted against the 2Θ angle, noted [2Θ] CuKα. Table 2 lists the instruments used for each sample analyzed.

Thermogravimetric analyses were carried out under argon flux on non-dried powdered M-S-H samples (30–40 mg) with a Mettler TGA 2 instrument (LMDC, Toulouse, France) and a heating rate of 10 °C/min from ambient temperature to 980 °C. The powders used for the test were obtained by crushing a cross section of the sample to 80 µm. The amount of Mg(OH)_2_ was quantified from the weight loss around 420 °C (335–455 °C) using the stepwise method [52] and calculated according to Equation (1):

(1) $${\mathrm{wt}\%\;\mathrm{brucite}}_{\mathrm{dry}}=\frac{\mathrm{water}\;\mathrm{loss}\;(\mathrm{brucite})}{{100-\mathrm{water}\;\mathrm{loss}}_{H_{2}O\;(25–550\;{}^{\circ}C)}}\;\times \;\frac{M_{\mathrm{brucite}}}{M_{H_{2}O}}\;\times \;100$$

where wt% brucite_dry_ corresponds to the weight percentage of brucite for 100 g of dry mass (g/100 g) and water losses are expressed in weight percentages. M_brucite_ is the molar mass of Mg(OH)_2_ (equal to 58.32 g/mol) and M_H2O_ is the molar mass of water (equal to 18.02 g/mol). The relative error on the brucite content was ±5–10% [52].

Solid chemical characterizations were carried out with an energy-dispersive spectrometry (EDS) system adapted to a scanning electron microscope (SEM; Hitachi S3500N, IRSN, Fontenay-aux-Roses, France). The system comprised two EDS Brücker SDD detectors working at 15 kV and a working distance of 16.8 mm. Observations were performed on a polished section (Protocol in Appendix A, Table A1) coated with a gold-palladium alloy (277 Å thick).

Microscopic imagery was carried out with the same instrument on fractures coated with a thin gold-palladium layer (92 Å thick) at 25 kV. The working distance depended on the sample and its geometry.

#### 2.3.2. Microstructure Analyses

**Figure 1:**
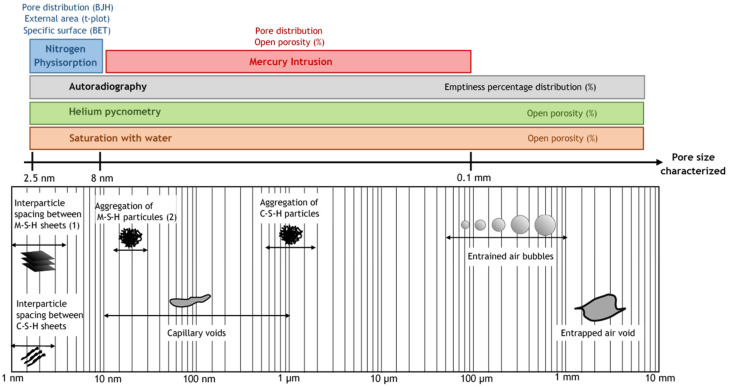
Representation of porosity characterization techniques as a function of pore range associated with the dimensional range of solids and pores in an M-S-H paste (adapted from Mehta and Monteiro [53]). (1) After Bernard [43] and (2) after Tonelli [46].

Different types of microstructure analyses were conducted. Figure 1 illustrates the porosity characterization techniques used as a function of pore range associated with the dimensional range of solids and pores in hydrated cement and M-S-H pastes. The porosity characterization of the pastes was carried out by combining several techniques. Pore distribution from 2.5 nm to 0.1 mm was determined by nitrogen physisorption and MIP. The open porosity of the samples was measured by autoradiography, MIP (Φ_M_), saturation of the material with water (Φ_W_), and by means of a calculation (Φ_H_) from the bulk (ρ_bulk_) and true densities (ρ_true_). X-ray microtomography was used to observe the distribution of unreacted silica particles in the pastes.

M-S-H samples were analyzed by X-ray microtomography using two different devices: SkyScan 1272 and SkyScan 1173 (Bruker microCT N.V, Fontenay-aux-Roses, France). A spatial resolution of 15 μm was obtained for a sample size of approximately 5 cm^3^. The parameters of the X-ray tube (100 kV, 100 mA) were set to optimize the contrast between the different phases and the pores. An angular step of 0.3° was used to acquire radiographs (total acquisition time of approximately 9 h).

Autoradiographic analyses were conducted on 1.5 cm × 1.5 cm × 2 cm samples made from colloidal silica. Samples were impregnated with C-14-methylmethacrylate (C-14-MMA) tracer solution, filling the connected porosity. The polymerization process was induced by gamma irradiation in order to fix the tracer to the open pore space. Samples were cut, polished and exposed on storage phosphor imaging plates (IPs) for 1–3 days. After exposure, the IPs were scanned using a FUJI-FLA 5100 digital scanner with a resolution of 1200 dpi and a 16-bit image depth to obtain the autoradiographs of the impregnated samples. The scanning resolution of the film was 10 µm. Quantification of porosity is based on the assumption that optical densities on autoradiographs are proportional to the content of the decaying tracer isotope in the material [54,55,56,57,58]. First, the optical densities of the autoradiographs are calculated. All intensity values or grey levels in the subdomains (pixels) are converted into the corresponding optical densities, which in turn are converted into activities with calibration curves measured for each exposure. Finally, the activities are converted into porosities by taking into account the beta attenuation and the true density of the samples (measured by helium pycnometry) [57]. Several PMMA standards of known C-14 activities are needed to convert the grey values of each pixel into porosity values [57,58,59,60]. The uncertainty of the C-14-PMMA autoradiography has been empirically determined to be approximately 10% [57]. Three samples with colloidal silica—one for every M/S—were analyzed of which one broke during impregnation (M/S = 1), making it impossible to analyze this ratio. The sample with M/S = 1.3 was divided into two parts, allowing for two analyses.

**Figure 2:**
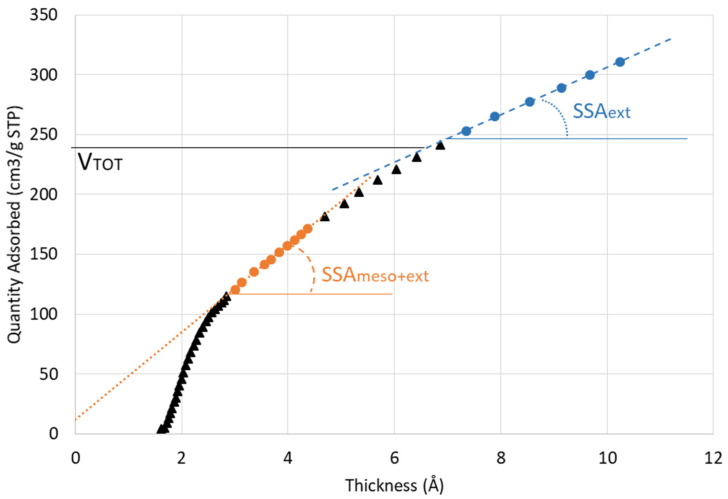
Illustration of the t-plot on an M-S-H paste highlighting the external surface area (SSA_ext_), the pore volume (V_TOT_), and the total specific surface area (SSA_meso + ext_), which corresponds to the specific surface area calculated with BET (after Galarneau et al. [61]).

Nitrogen sorption isotherms were measured using a Micromeritics 3Flex 3500 (Norcross, GA, USA) instrument equipped with version 4.05 of the 3Flex software (IRSN, Fontenay-aux-Roses). Prior to the analysis, the samples were reduced into pieces of approximately 0.2 cm^3^ and placed in a freeze dryer (Crios-50, Cryotec, IRSN, Fontenay-aux-Roses, France) for a least 5 days. They were then degassed (i.e., heated at 45 °C under a vacuum in a sample cell) in the instrument for 4 h to remove water and other physically adsorbed volatile material from the surface of the sample. The samples underwent nitrogen adsorption and desorption for approximately 20 h. Three measurements were carried out on each sample. The specific surface area (SSA_BET_) of the samples was calculated by applying the Brunauer-Emmett-Teller (BET) equation in the p/p^0^ range 0.05–0.2. External surface area (SSA_ext_) and total pore volume were calculated from the t-curve (obtained from the equations fitted on Aerosil 200 in [61]) for p/p^0^ < 0.8, between 7 and 10 Å on the second slope [61,62]. Figure 2 illustrates the t-curve of one sample with the slopes and calculation parameters. SSA_meso + ext_ corresponds to the mesopore + external surface areas and to the SSA_BET_. The pore distribution was obtained with the Barrett-Joyner-Halenda (BJH) method [63].

Mercury intrusion porosimetry was performed with an AutoPore IV 9500 porosimeter from Micromeritics (LMDC, Toulouse, France). The samples underwent the same pre-treatment as for nitrogen physisorption. After a pre-equilibrium step to fill the gaps between the sample and the chamber wall at 3 KPa, the pressures on mercury were automatically and stepwise raised to 413.69 MPa.

The determination of bulk densities (ρ_bulk_) was performed by measuring the pressure induced through the immersion of the sample in kerosene according to Archimedes’ principle, following the method initially proposed by Monnier et al. [64] and included in the AFNOR standard X31-505 [65]. The samples remained 4 h in kerosene before weighing.

Water content measurements were carried out at IRSN by weighing before and after heating at 105 °C until constant weight was reached. The water absorption of samples was determined by immersion in Milli-Q water for 24 h followed by heating at 105 °C until constant weight was reached. The open porosity (ϕ_w_) was determined with Equation (2):

(2) $$\phi_{W}=\frac{m_{\mathrm{water}}}{m_{\mathrm{total}}}\times \frac{\rho_{\mathrm{bulk}}}{\rho_{\mathrm{water}}}$$

where ρ_bulk_ is the bulk density of the material, ρ_water_ = 1 g/cm^3^, m_water_ is the mass of water in the saturated sample and m_total_ is the total mass of the sample after heating at 105 °C.

A pycnometer (Micrometrics, AccuPyc II 1340, IRSN, Fontenay-aux-Roses, France) was used to determine the true density (ρ_true_) of the samples. The volume of the samples of known mass was determined from the pressure change of helium in a calibrated volume. Measurements were performed in 10 cm^3^ modules on a set of pieces of 0.2 cm^3^. Fifteen cycles of introduction and extraction of helium were operated. The open porosity (ϕ_H_) was calculated with Equation (3):

(3) $$\phi_{H}=1\;-\frac{\rho_{\mathrm{bulk}}}{\rho_{\mathrm{true}}}$$

where ρ_bulk_ is the bulk density of the material and ρ_true_ is the true density of the material.

## 3. Chemical and Microstructural Characterization of M-S-H as a Function of Design and Curing Protocols

### 3.1. Influence of the Type of Silica

**Figure 3:**
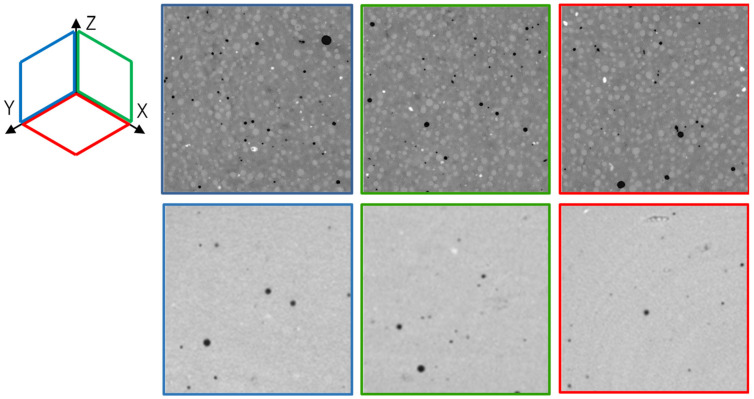
Tomography images: MS_1_SF_T50_21d (**top**) and MS_1_Cs_T20_3d (**bottom**).

**Figure 4:**
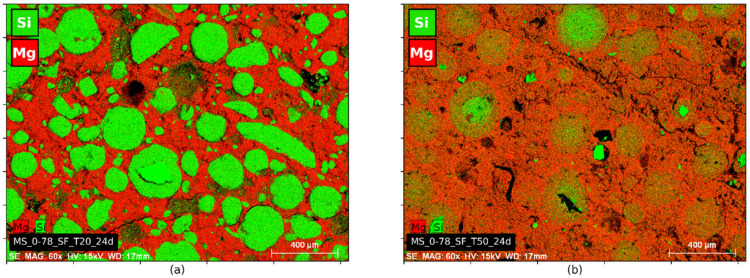
EDS images of M-S-H pastes with M/S = 0.78 and silica fume after 24 days of hydration. (**a**) curing at 20 °C; (**b**) curing at 50 °C.

**Figure 5:**
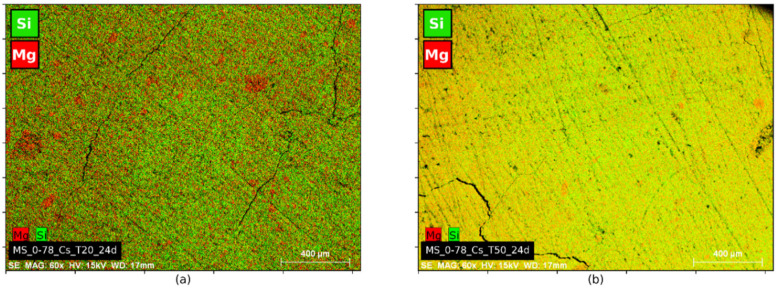
EDS images of M-S-H pastes with M/S = 0.78 and colloidal silica after 24 days of hydration. (**a**) curing at 20 °C; (**b**) curing at 50 °C.

Images of X-ray microtomography analyses (Figure 3) and EDS mappings (Figure 4 and Figure 5) showed that switching the silica source from silica fume to colloidal silica resulted in a better distribution of the particles and a higher homogeneity of the hydrated paste, irrespective of the curing temperature.

EDS mappings (Figure 4 and Figure 5) obtained from the pastes cured at 20 °C show the effect of the silica type on the size of the cluster of unreacted silica. The average cluster size was 200 µm for silica fume and 25 µm for colloidal silica. The latter resulted in a better dispersion of silica particles within the Mg-matrix, improving the homogeneity of the paste (even if this distribution does not allow to evaluate whether the dispersed silica has already reacted with MgO).

**Figure 6:**
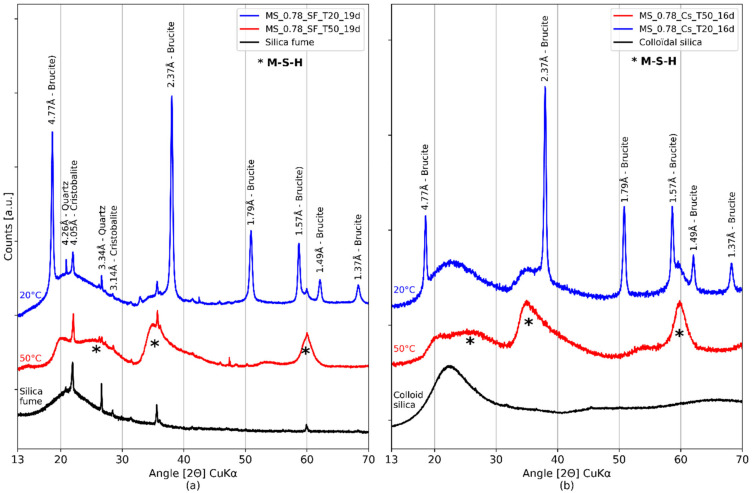
X-ray diffractograms of M-S-H pastes with M/S = 0.78. Comparison between the four protocols after two weeks of hydration. (**a**) Pastes with silica fume and M/S = 0.78 after 19 days of hydration at 20 °C (blue) or 50 °C (red) (**b**) Pastes with colloidal silica and M/S = 0.78 after 16 days of hydration at 20 °C (blue) or 50 °C (red).

Furthermore, the raw colloidal silica did not contain crystallized phases (such as quartz or cristobalite) but only amorphous silica. This was confirmed by the absence of the corresponding peaks (Table 3) on the X-ray diffractograms of colloidal silica pastes (Figure 6b), although quartz peaks were observed on the X-ray diffractograms of silica fume pastes (Figure 6a).

In conclusion, the use of colloidal silica led to improved homogeneity and purity, i.e., in terms of presence of M-S-H only, of the pastes.

### 3.2. Influence of the Curing Temperature

**Pastes prepared with silica fume.** Mg and Si EDS mapping of M-S-H pastes with M/S = 0.78 and silica fume are shown in Figure 4. The samples used for the tests originated from the same mix and were cured for 24 days at 20 °C or 50 °C.

Areas rich in silicon and poor in magnesium were observed for the 20 °C curing (Figure 4a). These areas correspond to residual unreacted silica fume clusters. At this temperature, the reaction of MgO with silica fume was slow and did not result in a homogeneous M-S-H paste at the time of observation. At 50 °C (Figure 4b), orange regions on the mapping correspond to a mix of Mg- and Si-containing phases, with an equivalent intensity of these elements. The matrix was enriched in silicon and the remains of the clusters were enriched in magnesium. This paste was more homogeneous than the paste cured at 20 °C.

In order to evaluate the mineralogical composition of the paste, XRD (Figure 6a) analyses were performed.

The sample cured at 20 °C contained brucite and amorphous silica (Figure 6a). The presence of M-S-H was detected but their formation was likely limited by the initial formation of brucite, at least at the tested ages [34,43]. For the 50 °C curing, no characteristic peak of brucite was visible and the amorphous silica seemed to have been consumed (the associated hump disappeared). Conversely, M-S-H humps were clearly observable (as M-S-H are a semi-amorphous phase, only broad peaks or humps are observed). The five M-S-H humps observed by Bernard et al. and Roosz et al. [33,43], including three main humps [32,46,66] (~20, **26**, **35**, 54, **60** [2Θ] CuKα) were present in the sample cured at 50 °C. The same observations were made for the other M/S pastes tested in the present study.

**Pastes prepared with colloidal silica.** Mg and Si EDS mapping of M-S-H pastes with M/S = 0.78 and colloidal silica are shown in Figure 5. As for the sample with silica fume, brucite was visible on X-ray diffractograms after 16 days of 20 °C curing (Figure 6b) while only M-S-H humps were present at 50 °C. The pastes with colloidal silica considered for this analysis were mechanically mixed, except for M/S = 1 for practical reasons (not produced at the same time). EDS mapping (Figure 5a,b) showed a better distribution of magnesium and silicon in the paste cured at 50 °C than in the paste cured at 20 °C. The clusters observed at 20 °C were reduced considerably at 50 °C, dropping from a diameter of 25 μm to a few microns.

In conclusion, the thermal curing at 50 °C resulted in a more homogeneous paste. Pastes showed a lower content of brucite at this temperature than pastes cured at 20 °C. No brucite was observed for the pastes with an M/S ratio equal to 0.78, even after only 16 days of curing for pastes designed with colloidal silica (Figure 6b).

To produce M-S-H with different M/S ratios, with properties comparable to those observed in synthesis (on powders) [32,33,43] and with the highest content of M-S-H and the lowest content of impurities, the suggested method is a protocol with colloidal silica and 50 °C thermal curing.

### 3.3. Formation of M-S-H

**Figure 7:**
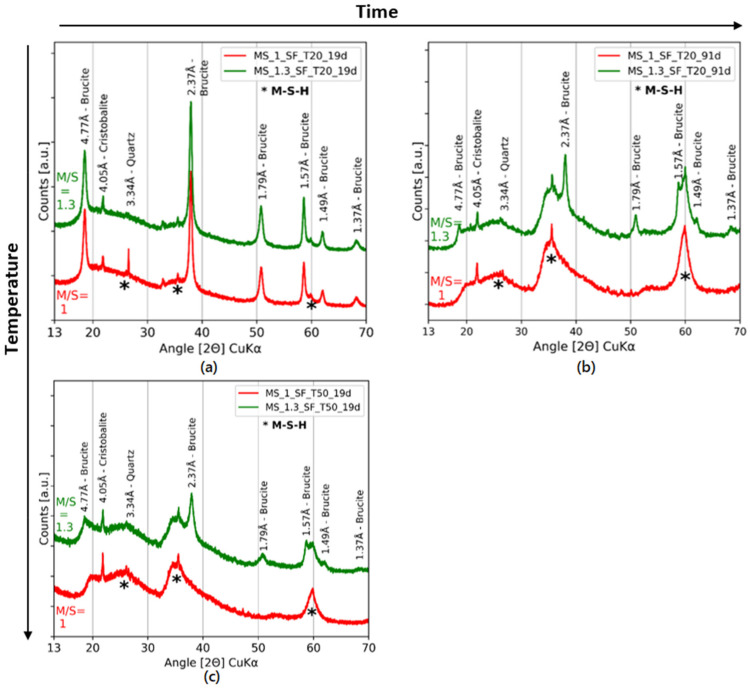
X-ray diffractograms of pastes with silica fume after 19 (**a**) or 91 days (**b**) with 20 °C curing, and with 50 °C curing after 19 days (**c**).

The kinetics of M-S-H formation at 20 °C, 50 °C and 70 °C have been studied on M-S-H powders by Bernard [51]. The purpose of this section is to compare the observations and conclusions between powders and pastes. The phenomena and kinetics of diffusion of ionic species—and the accessibility to water for reagents through already formed hydrate layers—are different between powders and cohesive pastes. Bernard et al. produced M-S-H powders by mixing magnesium oxide and silica fume with ultrapure water (water/solid ratio of 45) [43,51]. Figure 7 presents X-ray diffractograms of M-S-H pastes made with silica fume with M/S = 1 and M/S = 1.3 after 19 and 91 days of curing at 20 °C or 50 °C.

The precipitation of brucite was not impeded by curing at 50 °C for the two sources of silica. Brucite was observed on the XRD for M/S = 1.3 after 19 days of hydration at 50 °C (silica fume; Figure 7c). In addition, the M/S ratio seemed to influence the persistence of brucite. The higher the ratio, the longer the brucite remained present. Brucite was observed for high (M/S = 1.3) but not low (M/S ≤ 1) M/S ratios after either 19 days of thermal curing at 50 °C or 91 days at 20 °C (Figure 7).

This result was in agreement with the observations of Bernard on M-S-H powders [51]. In that study, no brucite was observed in M-S-H powders at M/S < 1.0 cured at 50 °C or 70 °C after the same hydration time (91 days), while at 20 °C some brucite was observed, indicating a faster formation of M-S-H at higher temperatures. For M/S ≥ 1.2, brucite was still observed even after one year at 50 °C and 70 °C. The presence of residual brucite was thus linked to the M/S ratio. The higher the M/S ratio, the longer the presence of brucite. It should be noted that TGA on pastes with colloidal silica no longer showed the presence of brucite after 5 months for all the M/S ratios in the present study. This result is presented in Section 4.2.

The effect of thermal curing could be twofold in that it may: (i) accelerate the dissolution of SiO_2_ and MgO and the diffusion of ionic species, and (ii) shift thermodynamic equilibria. Bernard [51] stated that temperature had a significant influence on the kinetics of M-S-H formation and their solubility, having observed a faster M-S-H formation at higher temperatures. The magnesium and silicon concentrations at 50 °C and 70 °C reached equilibrium with M-S-H faster than at 20 °C. According to Bernard’s work, the thermodynamic equilibrium of brucite was modified with the curing temperature. More Mg was needed in solution at 50 °C than at 20 °C to form brucite. Brucite only formed in the short term for lowM/S ratios with SiO_2_ dissolving and diffusing over time. The ratio in solution decreased, becoming more favorable to M-S-H and less to brucite (which dissolved).

In conclusion, the formation of M-S-H pastes from MgO and SiO_2_ (silica fume or colloidal silica) is a slow process that can be accelerated by thermal curing. As expected, the higher the M/S ratio, the longer the brucite is present.

## 4. In-Depth Characterization of Colloidal Silica M-S-H Pastes with Different M/S Ratios

As detailed in the previous section, the protocol with colloidal silica and a 50 °C curing yielded the highest content of M-S-H and the lowest content of other species. In this section, the samples analyzed originate from this protocol exclusively. Two types of mixing were tested, mechanical and manual. Three M/S ratios were studied: 0.78, 1 and 1.3. The pastes considered for Section 4.1 and Section 4.2 were MS_0-78_CS_T50_M, MS_1_CS_T50_E and MS_1-3_CS_T50_M.

### 4.1. Morphology of M-S-H

**Figure 8:**
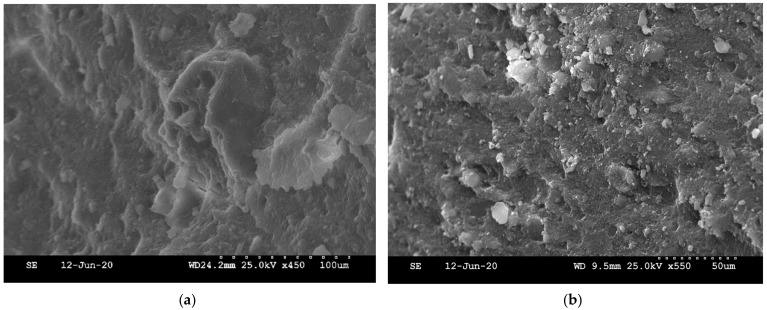

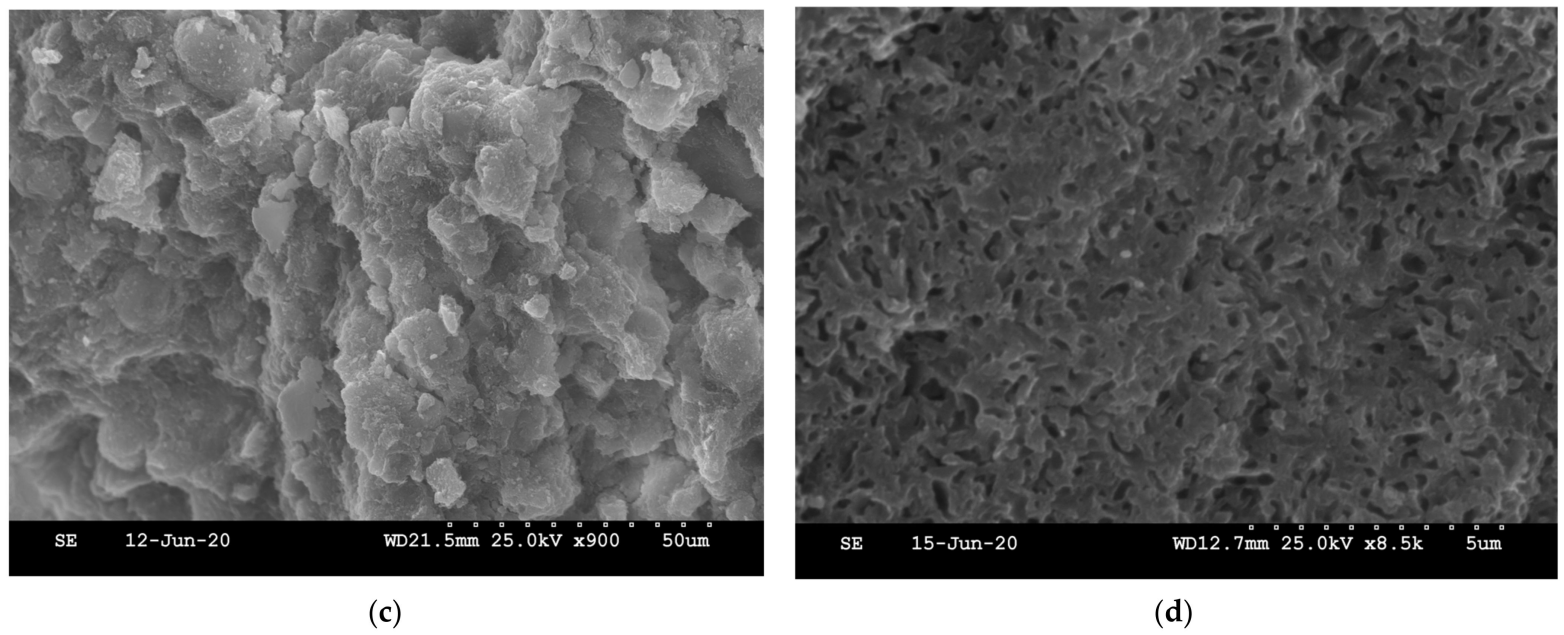
Fresh facture images on SEM of colloidal silica pastes with a 50 °C curing after 4.5 months of hydration: (**a**) and (**c**) M/S = 1.3, (**b**) M/S = 0.78 and (**d**) M/S = 1.

The morphology of M-S-H pastes was observed by SEM on fractured samples (Figure 8), proving to be similar to the morphology reported by Tonelli et al. [46]. M-S-H did not show crystalline facies but rather aspects of gel with globular chains. M-S-H showed high porosity at high magnification (Figure 8c), as we will discuss in Section 4.3.

### 4.2. Solid Composition

**Figure 9:**
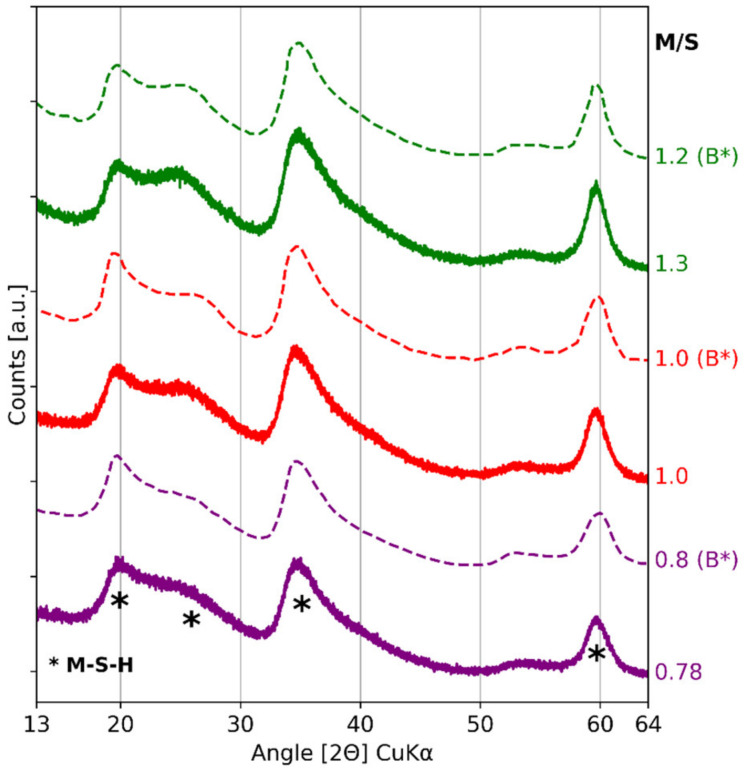
X-ray diffractograms of three M-S-H pastes with colloidal silica after 4.5 months of a 50 °C curing and three M-S-H powders synthetized by Bernard et al. [43] (dashed lines, noted B*). Manual mixing was used for pastes M/S = 0.78 and M/S = 1.3; mechanical mixing was used for M/S = 1.

X-ray diffractograms of three M-S-H pastes are presented in Figure 9. The humps of M-S-H (see Table 3 for positions) were observed on all diffractograms [32,46,66]. No brucite peaks were identifiable. The proportion of second broad reflection (~26 [2Θ] CuKα) relative to the rest of the diffractogram increased along with the M/S ratio, as was the case for diffractograms of M-S-H powders of Bernard (dashed lines in Figure 9) [43]. The first broad reflection (20 [2Θ] CuKα) simultaneously decreased, indicating a rearrangement of the structure as the M/S ratio increased. As proposed by Bernard, the first broad reflection would be linked to a structure similar to talc (a natural analogue of M-S-H) while the second broad reflection would be linked to serpentine. Thus, the X-ray diffractograms indicated a rearrangement of the structure with the increase in M/S, from a talc-like structure to serpentine, as Nied et al. observed in 2016 [32].

**Figure 10:**
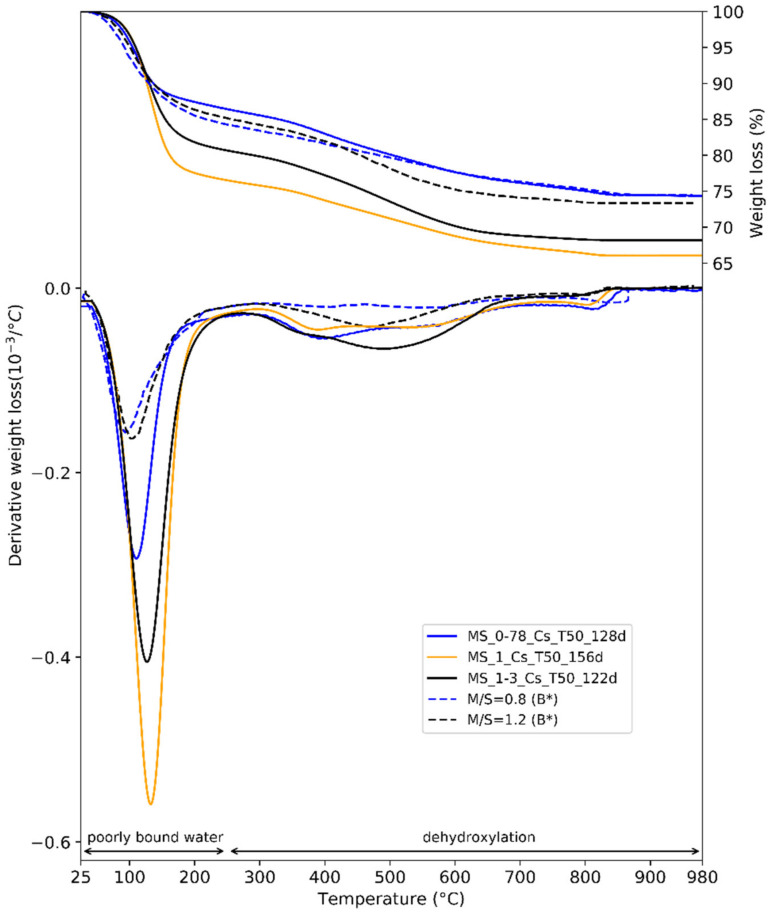
TGA results of three M-S-H pastes with colloidal silica after 4.5 months of a 50 °C curing and two M-S-H powders synthetized by Bernard et al. [43] (dashed lines, noted B*). Manual mixing was used for pastes M/S = 0.78 and M/S = 1.3; mechanical mixing was used for M/S = 1.

The TGA curves of M-S-H pastes and the derivative weight losses are shown in Figure 10. Brucite (400–420 °C) was absent. The three water loss regions expected for M-S-H were observable. The first water (30–280 °C) loss region was linked to H_2_O (poorly bounded) loss in M-S-H.

The second and third water losses have been attributed to silanol and/or magnesium hydroxyl groups in M-S-H, but they may also comprise water present as a monolayer on the M-S-H surface [32,43,52]. Their attribution is controversial. Table 4 recaps the views and assumptions of various authors. The structure of M-S-H has been compared to that of many phyllosilicates (sepiolite, talc, etc.) [32,33,35,38,43]. Nied et al. [32] suggested that the weight loss between 280 °C and 750 °C corresponds to hydroxyl groups with Mg^2+^, while the one between 750 °C and 840 °C would be linked to silanol groups on M-S-H. In the case of synthetic talc, Dumas et al. [67] attributed the weight loss (150–450 °C) to silanol (Si–OH) and magnesium (Mg–OH) hydroxides on the sheet edges. The next loss (450–750 °C) would be linked to the dehydroxylation of small particles, whereas the high temperature loss (750–850 °C) would correspond to the dehydroxylation of bigger particles and the formation of enstatite and silica. For Bernard et al. [43], and according to the work of Zhuravlev [68] on amorphous silica, the weight loss around 390 °C (observable IN Figure 10 on curves M/S = 0.78, 0.8 and 1) would match the dehydroxylation of silanol groups (on the surface) while the weight loss at 500 °C would be related to dehydroxylation of Mg–OH in M-S-H. Moreover, the removal of internal OH groups of silanols happened at around 900 °C.

The position shared by the authors quoted is the following: (i) the weight losses between 200 °C and 800 °C for M-S-H systems are linked to the dehydroxylation of a hydroxyl group in M-S-H, and (ii) the potential offsets of the losses are linked to the evolution of the structure of M-S-H. With regard to the type of hydroxyl groups associated with each temperature, the most recent and documented assumption (Bernard et al. [43]) was chosen.

The second water loss region between 280 °C and 750 °C differed with the M/S ratio (Figure 10). For M/S = 0.78 and M/S = 1, the second hump was located in the same place, while for M/S = 1.3 the second hump was shifted to the right. The offset of the hump (400–500 °C) resulting from the increase in M/S from 0.78–1 to 1.3 also appeared on the TGA on M-S-H powders from Bernard et al. and Nied et al. [32,43]. The height of the last water loss region between 750 °C and 840 °C also evolved with the M/S. The amount of bound water increased along with the magnesium content. As the weight loss around 400 °C has been associated to surface Si–OH and the weight loss around 500 °C to Mg–OH in M-S-H [43], the offset of the hump would indicate the formation of more Mg–OH groups with the increase in M/S. For M/S = 1.3, the broader weight loss around 500 °C covered the weight loss around 400 °C of M/S = 0.78 and M/S = 1, indicating that the amount of surface Si–OH groups remains stable. Conversely, the decrease in height of the weight loss peak around 800 °C as M/S increases would indicate a decrease in the internal Si–OH content of M-S-H. Thus, the internal structure of M-S-H would evolve with M/S.

The structure of M-S-H has been compared to that of many phyllosilicates (sepiolite, talc, etc.) and characterized as a sheet structure evolving with the M/S [32,33,35,38,43]. The silicate sheets would be linked by layers of octahedral-coordinated Mg^2+^ ions [32], with water present both as adsorbed water and in structure as H_2_O and hydroxyl groups [43]. As the X-ray diffractograms (Figure 9) illustrate, the interlayer distance decreases while M/S in M-S-H increases [43], as is the case for C-S-H when the calcium to silicon (C/S) ratio increases [69]. According to the TGA results (Figure 10), this reduction of the interlayer distance would be associated with the formation of Mg–OH in M-S-H, with internal silanol groups being reduced. The evolution mechanism of the M-S-H structure with the variation of M/S would then be similar to that of the C-S-H with the variation of C/S. A possible parallel could be inferred between the Ca–OH that forms between the chains of Ca–Si and the Mg–OH that would form between the layers of Mg–Si as C/S or M/S increase.

The two analyses allowed to describe the pastes as composed only of M-S-H. The evolution of the structure with M/S appeared in the XRD and TGA. Talc-like characteristics were observed at low M/S, while serpentine-like reflections were associated with high M/S. Moreover, we detected an increase in the Mg–OH content of M-S-H as M/S increased.

### 4.3. Microstructure and Porosity

Several techniques were used to characterize the microstructure and porosity of M-S-H pastes. Figure 1 illustrates the panel of porosity characterizations associated with the dimensional range of solids and pores in an M-S-H paste. In this section, we propose the characterization of the porous structure starting from the smallest (physisorption) to the largest scale (water saturation). Since the mixing protocol and the w/b could influence the structure and porosity of a paste, the tests were carried out on both pastes with manual (noted M, w/b~1.45) and mechanical (noted E, w/b~1.1) mixing. The results presented correspond to pastes MS_0-78_CS_T50_M/E, MS_1_CS_T50_E, and MS_1-3_CS_T50_M/E.

**Figure 11:**
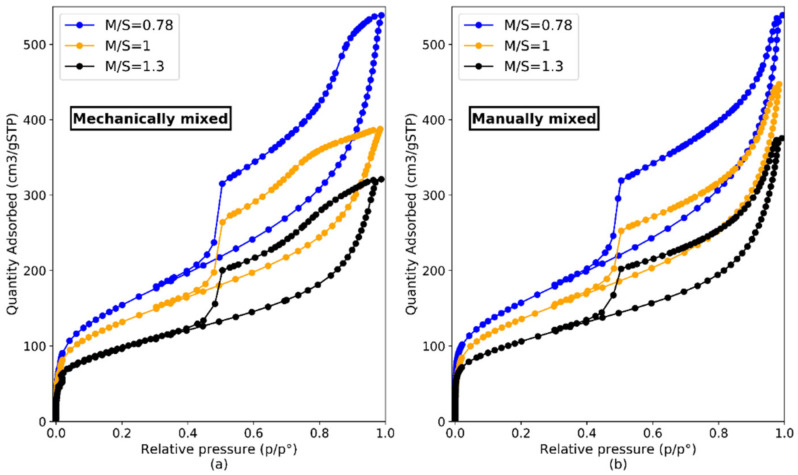
N_2_ adsorption-desorption isotherms of M-S-H samples with M/S = 0.78, M/S = 1.0 and M/S = 1.3, mixed mechanically (**a**) or manually (**b**).

Figure 11 shows the N_2_ adsorption-desorption isotherms of M-S-H samples made with M/S = 0.78, M/S = 1.0 and M/S = 1.3, mixed manually (Figure 11b) or mechanically (Figure 11a). The mixing protocol and the w/b seemed to influence the form of the isotherm at relative pressures > 0.7 (for both the adsorption and desorption curves). The mechanical mixing—associated to a lower w/b—resulted in a smoother desorption for very high relative pressures and a lower adsorbed quantity. In the range p/p^0^ = {0; 0.7} the mixing process and w/b had no influence, probably due to the creation of “bigger” pores with manual mixing (where the w/b was higher). The internal structure of M-S-H remained the same since low pressures were not affected.

**Figure 12:**
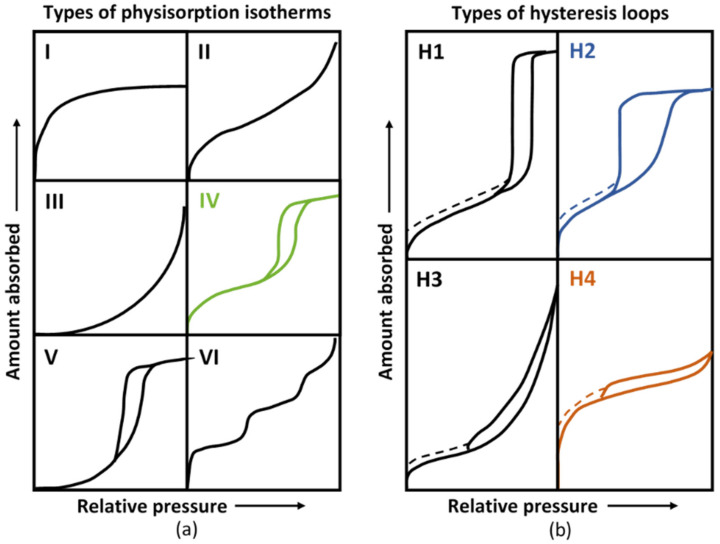
Types of physisorption isotherms (**a**) and hysteresis loops (**b**) (after Sing [70]).

Figure 12 illustrates the different types of isotherms and hysteresis loops following the IUPAC classification [70]. The isotherms presented a hysteresis loop characteristic of a Type IV isotherm according to this classification. The adsorption part of the Type IV isotherm could be attributed to a monolayer-multilayer adsorption. The initial vertical slope at low p/p^0^ was characteristic of the presence of microporosity (equivalent diameter between 0.4 nm and 2 nm).

Hysteresis has been associated with capillary condensation taking place in mesopores (2–50 nm in diameter) [43]. M-S-H pastes made by mechanical mixing (lowest w/b), exhibited hysteresis comparable to H4 types with a small portion of the curve (on desorption at high p/p^0^) which would correspond to H2 type. The Type H4 loop is associated with narrow slit-like pores while the Type H2 is harder to interpret and is often associated with pore size and a not well-defined shape [70]. The form of the high pressure hysteresis for mechanically mixed pastes could therefore relate to the presence of specific bottleneck-shaped pores [71]. Overall, the structure we observed corresponded to plate-like materials with mesopores. The shape of the isotherms—especially of manually mixed pastes (highest w/b)—resembled that of synthetic saponite-like materials prepared by traditional hydrothermal crystallization at 513 K with H_2_O/SiO_2_ = 50 by Bisio et al. [72]. The form of the hysteresis was also similar to that of the M-S-H powders synthesized by Bernard [43] under hydrothermal treatment (180 °C for 4 days).

According to Figure 11, the M/S ratio of M-S-H pastes appeared to influence the amount of N_2_ adsorbed: the lower the M/S, the higher the total N_2_ adsorbed.

**Figure 13:**
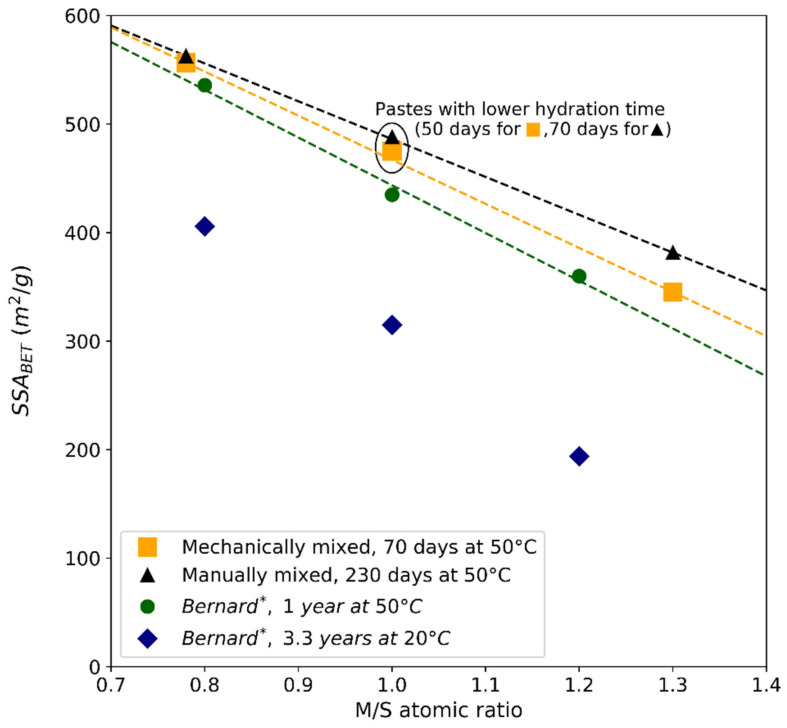
Specific surface area (SSA_BET_) found in M-S-H pastes as a function of the M/S atomic ratio and of the mixing protocol. The dotted lines are the trend lines from the mechanically mixed (orange), manually mixed (black) and * Bernard et al. [43] measurements (green: M-S-H powders equilibrated for 1 year at 50 °C; blue: M-S-H powders equilibrated for 3.3 years at 20 °C).

The specific surface areas calculated with BET (SSA_BET_) on M-S-H pastes hydrated from 2 (mechanically mixed) to 7.5 months (manually mixed; M/S = 1 is an exception, as it was hydrated for 2.5 months) are displayed in Table 5. Figure 13 illustrates the evolution of the specific surface as a function of the M/S ratio and the mixing protocol. Bernard’s data obtained on synthetic powders [43] are plotted in blue and green.

High SSA_BET_ were close to large surface areas reported by Bernard [43] for powders with a similar curing (1 year at 50 °C). As expressed by Bernard, specific surfaces were much lower for samples cured for 3.3 years at 20 °C due to a longer hydration time. Over time, the porous network could evolve. Initially, magnesium phases are formed upon contact between water and magnesium oxide and/or silica. As is the case with inner C-S-H [73], denser M-S-H could gradually be formed in the inner layer of reacted silica fume clusters, which would result in finer porosity, lower specific surface areas and lower pore volumes. In the case of M-S-H pastes with colloidal silica, no silica clusters were formed, reducing the possibility of a more dense M-S-H formation.

Contrary to Bernard’s hypothesis, according to which M-S-H forms in non-diluted systems or might exhibit a smaller SSA in field experiments, the experiments presented in this paper proved that M-S-H pastes showed similar specific surfaces to powders, both cured at 50 °C. The specific surface seemed independent of the mixing protocol for low M/S ratios (0.78 and 1). The SSA_BET_ decreased with the M/S ratio in M-S-H with a slightly different slope from that of Bernard’s data. At high M/S (M/S = 1 and M/S = 1.3), a gap arose between the data of Bernard, the data for manual mixing (w/b = 1.45) and the data for mechanical mixing (w/b = 1.1). The measurement uncertainty and the standard deviation (obtained by repeating the test) are too low to explain the difference in value between the protocols for these M/S values. High M/S ratios seem to be more affected by curing time and type of mixing. As for the effect of the mixing protocol at high M/S—highlighted in Section 3.2.—the high content of MgO compared to SiO_2_ implies a slower consumption of brucite. This could explain a different low-scale organization of the material between the pastes, following the initial brucite content (brucite is a crystallized phase, as opposed to M-S-H). The consumption of brucite to form M-S-H in a hardening shape could produce a different porous network.

**Figure 14:**
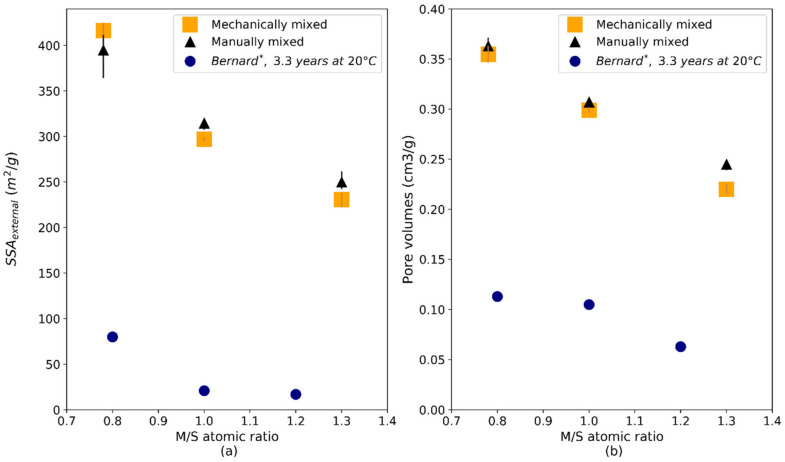
SSA_ext_ (**a**) and pore volumes (**b**) obtained by t-plot in M-S-H pastes as a function of the M/S atomic ratio and of the mixing protocol. * Bernard et al. measurements (in blue) correspond to M-S-H powders equilibrated for 3.3 years at 20 °C.

Pore volumes and SSA_ext_ are plotted in Figure 14. As was the case with SSA_BET_, the values measured were higher than those of Bernard on powders equilibrated for 3.3 years at 20 °C [43]. No value was presented by Bernard on samples equilibrated for 1 year at 50 °C. SSA_ext_ and pore volumes were notably high for all our samples, implying a high content of micro- and mesopores. A clear trend was visible with the evolution of the M/S ratio. An increase in M/S implied a decrease in external surface area and pore volume. The porosity of samples with high M/S ratios (M/S = 1.3) seemed to contain less micro- and mesopores than the porosity of samples with low M/S ratios (M/S = 0.78).

**Figure 15:**
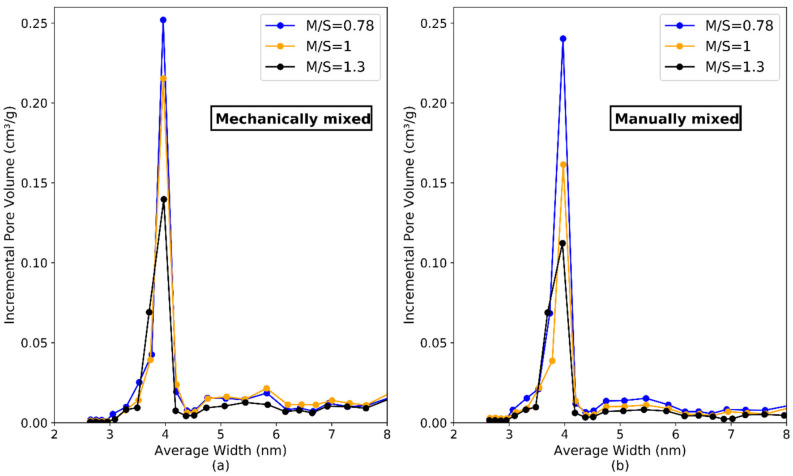
Pore size distribution by N_2_ physisorption of M-S-H samples with M/S = 0.78, M/S = 1.0 and M/S = 1.3, mixed mechanically (**a**) or manually (**b**).

The pore size distribution between 2.5 and 8 nm—according to the BJH method—is shown in Figure 15. All samples showed a peak around 4 nm. The incremental pore volume (cm^3^/g) increased as the M/S atomic ratio decreased, confirming that the M-S-H pastes with high M/S contained less micro- and mesopores than the pastes with lower M/S. The common location of the peak and the similar appearance of the curves indicated that the mixing protocol and the w/b did not influence this range of pores.

**Figure 16:**
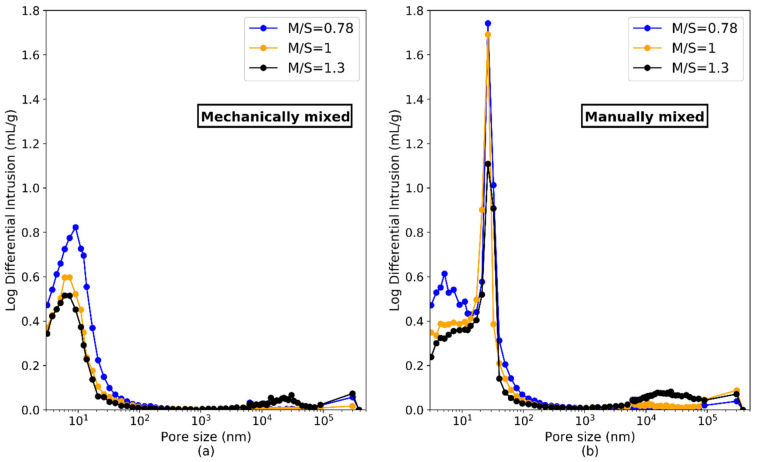
Pore size distribution by mercury intrusion porosimetry (MIP) of M-S-H samples with M/S = 0.78, 1.0 and 1.3, mixed mechanically (**a**) or manually (**b**).

In order to characterize the pore size distribution at the higher scale, MIP was used. The pore size distribution between 8 and 4 × 10^5^ nm is shown in Figure 16. The pastes manually mixed showed a family of small pores between 8 and 10 nm with a high peak centered at 26 nm, while the mechanically mixed pastes only exhibited a family of pores with a mean value centered at 9 nm. A manual mixing associated to a high w/b (1.45) therefore influenced the size and distribution of the mesopores by creating supplementary capillary pores (26 nm in diameter). The porosity values (Table 6) obtained with MIP will be discussed in the following paragraphs.

**Figure 17:**
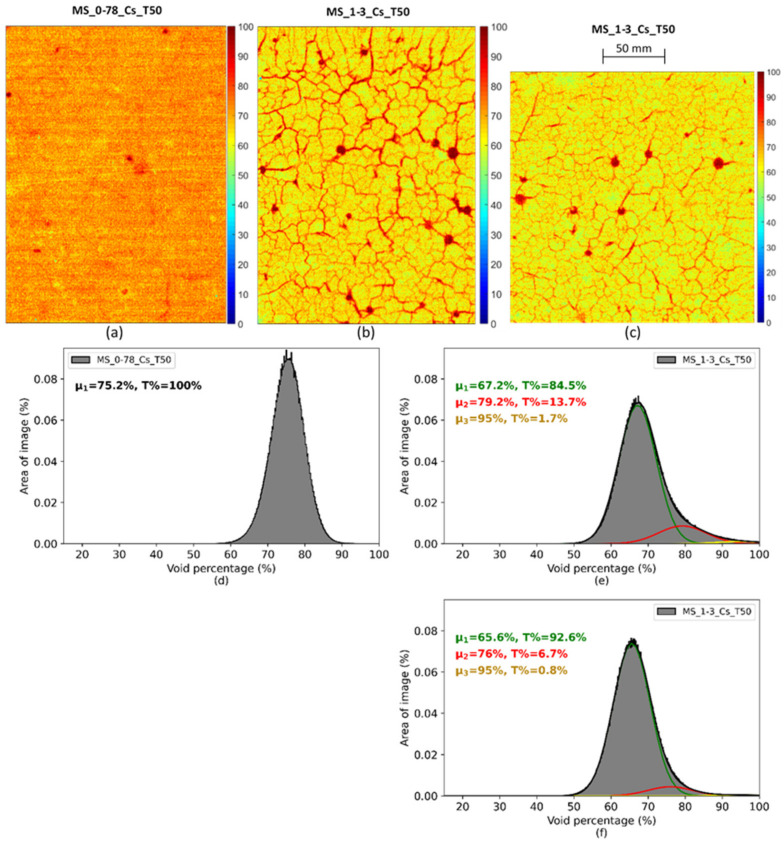
Autoradiography maps of M-S-H manually mixed pastes (**a**) M/S = 0.78; (**b**,**c**) M/S = 1.3 and associated porosity histograms and deconvolution—(**d**) for (**a**,**e**) for (**b**,**f**) for (**c**). µ is the mean of the phase and T% corresponds to the percentage of the phase into the total histogram.

Autoradiography was useful for establishing a link between the pore distribution and the overall porosity of the material, since it provides a 2D image of void percentage on samples and a histogram of the void percentage distribution. Autoradiography analyses were carried on three samples (one for M/S = 0.78 and two for M/S = 1.3). Figure 17a–c show the autoradiography maps. A cracking phenomenon—similar to shrinkage—appeared in the samples (Figure 17b,c) as soon as water was removed from them by drying, vacuum, etc. This experimental perturbation did not prevent us from analyzing the porosity of the samples through the extraction of cracks and bubbles by deconvolution of the porosity histograms. The histograms associated with the maps (Figure 17d–f) show the distribution of the void ratio (porous areas and cracks). In the histograms—where all kinds of features were taken into account—the highest values approximately reach 100%. For M/S = 1.3, three Gaussian distributions were used to reproduce the histogram. For M/S = 0.78, only one Gaussian distribution was necessary. The characteristics of the Gaussian distributions are displayed in the histogram (Figure 17d–f). For M/S = 1.3, the first Gaussian distribution with µ = 58% (sample 1) or µ = 56.4% represented more than 80% of the histogram. The two other Gaussian distributions—with µ = 72% and µ = 89% (sample 1) or µ = 68.9% and µ = 85%—would correspond to bubbles and cracks. The removal of cracks corresponded to considering only the first Gaussian for each histogram. The average value of the porosity obtained by further treatment would then be 57.2% for M/S = 1.3 and 67.5% for M/S = 0.78.

The M/S-dependent effect on cracking was observed in the autoradiography maps. As explained in Section 4.3 and observed in Figure 17, the sample with M/S = 1.3 showed numerous bubbles and cracks. The sample with M/S = 1 cracked during impregnation while the samples with M/S = 0.78 did not show large cracks. According to Figure 15, the samples in question did not exhibit any difference in low-scale pore distribution, except for the amount of N_2_ adsorbed. Thus, the difference in the cracking pattern was not due to the finer distribution of the pores. While the pastes with the lowest M/S (M/S = 0.78) were the most porous, they underwent the least cracking. Preferential and faster cracking appearing for high M/S could therefore be linked to lower mechanical properties of the material.

Autoradiography and MIP provided a first idea of the total open porosity of the material. Other measurements were carried out in order to obtain a clearer picture of the porosity of the pastes (Table 6). The lowest porosity value was obtained with MIP, followed by the values acquired with helium pycnometry and kerdane, autoradiography and water porosity. These observations can be explained by the range and type of pores considered depending on the test.

MIP explored only the connected porosity; while pores > 8 nm were taken into account, large defects (air bubbles and very open cracks) were ignored. The porosity values obtained by helium pycnometry correspond to the open porosity, including cracks and bubbles. Autoradiography measured the connected porosities, with air bubbles and cracks being removed during post-processing (smallest visible pores = 10 μm). Water porosity took into account the connected porosity, bubbles and cracks. The latter is therefore the largest measured value. Moreover, the 105 °C drying protocol used for water porosity may consume certain hydrates, resulting in a potential small overestimation of the actual porosity. As each method had its limitations, it was interesting to combine them all to characterize the material.

A general trend could still be observed. Manually mixed pastes had a slightly higher porosity (10% more) than mechanically mixed ones (these results were consistent with the higher w/b ratio used for manual mixing). Overall, the porosity of these pastes was high. The influence of the M/S ratio on the overall porosity was difficult to assess because the pastes were very porous (due to the high w/b ratio imposed). C-S-H pastes cast with a similar protocol (colloidal silica) also have a high porosity, close to 60% according to Kangni-Foli [74]. The effect of mixing and w/b disappeared only on a smaller scale. According to Bernard [75], the molar volume of M-S-H increases with increasing M/S. It is expected that the higher the solid molar volume, the lower the pore volume [76]. This observation can be found in our study through N_2_ physisorption and MIP. The more the M/S increases, the more the absorbed amounts of N_2_ and Hg and the specific surface area decrease, thus indicating a higher mesoporosity. On a larger scale, the porosities measured by the different techniques show this same trend. The pastes were mainly mesoporous, with a small amount of micropores and some defects appearing on a larger scale (bubbles due to mixing and cracks due to pre-treatments).

The microstructure of M-S-H pastes was characterized in this study. On a smaller scale, at the level of silica sheets (Figure 1), physisorption analyses described the material as a highly porous phase with plate-like micropores and mesopores. The increase in M/S implied a decrease in the quantity of N_2_ adsorbed, as well as a reduction of the specific surface (SSA_BET_), the SSA_ext_ and the micro- and mesopore volume. At the level of capillary pores to clusters of globules of M-S-H (Figure 1), MIP was useful for distinguishing the effect of the mixing protocol. Mechanically mixed pastes exhibited a mesopore family around 10 nm, while manual mixing also created a high amount of mesopores around 30 nm. The mechanical mixing—which lasted longer and used a higher speed and strength—helped reduce the w/b, leading to a denser paste with less porosity. On a larger scale, porosity measurement protocols provided information about open porosity. On autoradiography maps, we observed the formation of cracks as soon as the water was removed from the samples. This mechanism highlighted the mechanical strength and sensibility of M-S-H in a relatively humid environment. The removal of cracks and bubbles from histograms of porosity showed the high open porosity of the pastes (approximately 60% for manually mixed pastes). This high value was supported by other characterization methods, although the porosity measurements were influenced by pre- or post-treatment of the samples. Drying at 105 °C after water saturation and bulk density measurements could lead to the calcination of hydrates, resulting in an overestimated porosity. Overall, the open porosity of M-S-H pastes could be considered to be 70% for manually mixed pastes and 62% for mechanically mixed pastes, with this percentage increasing as the M/S ratio decreases.

## 5. Conclusions

This paper focused on the chemical and microstructural properties of cohesive M-S-H pastes. Four protocols to obtain cohesive and homogenous M-S-H pastes—without brucite or other species—were tested in this study. The increase in curing temperature (20–50 °C) accelerated the consumption of brucite and favored the formation of M-S-H, with colloidal silica enabling a better distribution of particles than silica fume. The higher the M/S ratio, the longer the brucite was present. The protocol of choice for M-S-H manufacturing therefore involves colloidal silica and MgO with w/b = 1 and mechanical mixing (140–285 rd/min rotation for 10 min), followed by a thermal cure at 50 °C for 50 days (minimum recommended).

X-ray diffraction and thermogravimetric analyses indicated a rearrangement of the structure—from talc-like structure to serpentine—as M/S increased, linked to a decrease in the interlayer distance and an increase in the Mg–OH content in M-S-H. The evolution of the M-S-H structure would be similar to that of the C-S-H. A parallel could be drawn between the Ca–OH that forms between the chains of Ca–Si and the Mg–OH that would form between the layers of Mg–Si as C/S or M/S increase.

The porosity of M-S-H pastes was studied from the level of entrained air bubbles and voids to the level of silica sheets. On a large scale, the combination of different characterization methods made it possible to quantify the total, open porosities of the pastes. Mechanical mixing reduced the open porosity from 70% (manual mixing) to 62%. This value changed along with the M/S ratio in that the higher the ratio, the lower the porosity. On a smaller scale—from the capillary pores to the level of clusters of M-S-H globules—manual mixing resulted in an additional family of pores (around 30 nm) that mechanically mixed pastes did not have, the latter only showing a family of mesopores centered on 10 nm. The finer porosity of the mechanically mixed pastes can be explained by the reduction in w/b due to increased mixing speed and power, which leads to denser and less porous pastes. On the smallest scale studied in this article (silica sheets), it seemed that the specific surface area and the amount of porosity increased when the M/S ratio decreased, while the size of the pores remained unaltered. M-S-H comprise a highly porous phase with plate-like micro- and mesopores and are sensitive to changes in humidity.

## Figures and Tables

**Table 1:** Mix proportions. Sp/b represents the mass percentage of superplasticizer against binder.

Type of Silica	M/S	T (°C)	Sample Name	g				Sp/b	w/b
				MgO	Silica Fume	Milli-Q-Water	Superplasticizer (Dry Extract)		
Silica fume	0.78	20	MS_0-78_SF_T20	176.4	336.9	513.8	0	0	1
		50	MS_0-78_SF_T50						
	1	20	MS_1_SF_T20	208.7	309.2	522.2	0	0	1
		50	MS_1_SF_T50						
	1.3	20	MS_1-3_SF_T20	242.8	278.1	526.3	0	0	1
		50	MS_1-3_SF_T50						
**Type of Silica**	**M/S**	**T (°C)**	**Sample Name**	**g**				**Sp/b**	**w/b**
				**MgO**	**Rheomac AS 150**	**Milli-Q-Water**	**Superplasticizer (Dry Extract)**		
Colloidal silica	0.78	20	MS_0-78_Cs_T20_M	63.2	241.3	145.8	8.94	4.9%	1.45
		50	MS_0-78_Cs_T50_M						
	0.78	50	MS_0-78_Cs_T50_E	241.1	920.3	312.5	47.4	6.8%	1.1
	1	50	MS_1_Cs_T50_M	71.7	213.3	151.5	9.18	5.1%	1.45
	1	20	MS_1_Cs_T20_E	292.5	872.7	292.7	45.1	6.2%	1
		50	MS_1_Cs_T50_E						
	1.3	20	MS_1-3_Cs_T20_M	119.8	274.3	235.1	7.14	2.8%	1.45
		50	MS_1-3_Cs_T50_M						
	1.3	50	MS_1-3_Cs_T50_E	331.7	760.8	403	38.25	5.4%	1.1

**Table 2:** Protocol used on samples in X-ray diffraction.

Sample Name	Location of Test	Protocol
MS_0-78_SF_T20_19d	Sorbonne Université	PANalytical X’Pert Pro (PANalytical Empyrean instrument) operating at 40 kV and 40 mA with a Cu anti-cathode (λ~1.54 Å)
MS_0-78_SF_T50_19d	Sorbonne Université	
MS_0-78_Cs_T20_16d	Sorbonne Université	
MS_0-78_Cs_T50_16d	Sorbonne Université	2Θ = 5°–70° with a step size of 0.0131, for a total duration of 4 h
MS_1_SF_T20_91d	Sorbonne Université	
MS_1-3_SF_T20_91d	Sorbonne Université	
Colloidal silica	LMDC	
Silica fume	IRSN	
MS_1_SF_T20_19d	IRSN	
MS_1-3_SF_T20_19d	IRSN	PANalytical Aeris operating at 600 W, 40 kV and 15 mA, with a Cu anti-cathode (λ~1.54 Å),
MS_1_SF_T50_19d	IRSN	
MS_1-3_SF_T50_19d	IRSN	
MS_0-78_Cs_T50_128d	IRSN	step size of 0.0109, for a total duration of 20 min
MS_1_Cs_T50_156d	IRSN	
MS_1-3_Cs_T50_122d	IRSN	

**Table 3:** Indexing of X-ray peaks for quartz, cristobalite, brucite, amorphous silica, and M-S-H.

References	Species	Unit	Peaks Indexing					
COD 96-901-0145 and COD 96-900-9687	Quartz and cristobalite	Å	4.26	4.05	3.34	3.14		
		[2Θ] CuKα	20.85	21.92	26.65	28.39		
Bernard et al. [36]	Brucite	Å	4.76	2.74	2.37	1.79	1.57	1.50
		[2Θ] CuKα	18.6	32.7	38	50.9	58.7	62
Data of Figure 4	Amorphous silica	Å	4.04					
		[2Θ] CuKα	22					
Bernard et al. [43] and Roosz et al. [33]	M-S-H	Å	4.43	3.42	2.56	1.70	1.54	
		[2Θ] CuKα	20	26	35	54	60	

**Table 4:** Different attributions of weight losses in TGA for M-S-H or correlated phases.

Author	Species Concerned	Temperature and Phase Associated to Weight Losses		
Lothenbach et al. [52]	M-S-H	270–700 °C		
		hydroxyl groups in M-S-H		
Nied et al. [32]	M-S-H	280–750 °C		750–840 °C
		Mg–OH in M-S-H		Si-OH in M-S-H
Dumas et al. [67]	talc	150–450 °C	450–750 °C	750–850 °C
		Mg–OH and Si–OH	small particles	big particles
Zhuralev [68]	amorphous silica	190–400 °C	400–900 °C	900–1200 °C
		vicinal bridged OH groups of silanols	geminal OH groups of silanols	complete removal of all OH groups
Bernard et al. [43]	M-S-H	390 °C	500 °C	900–1200 °C
		surface Si–OH in M-S-H	Mg–OH in M-S-H	internal Si–OH in M-S-H

**Table 5:** Average specific surfaces and volume calculated for M-S-H pastes (colloidal silica and thermal cure at 50 °C) using BET and t-plot methods from nitrogen isotherms.

	Mechanically Mixed			Manually Mixed		
M/S	0.78	1	1.3	0.78	1	1.3
w/b	1.1	1	1.1	1.45	1.45	1.45
Age (days)	70	50	70	230	70	230
Average specific surface area BET (m^2^/g)	556.7	475.2	345.3	562.9	488.2	381.6
Medium uncertainty	1.2	0.8	1.1	0.8	1.1	0.6
Standard deviation	11.7	3.7	6.6	8.7	8.1	3.0
External average specific surface area (t-plot) (m^2^/g)	416.6	296.8	230.6	394.9	314.6	249.8
Standard deviation	6.5	1.8	7.4	26.3	5.4	9.5
V_tot_ (cm^3^/g)	230.0	193.5	142.3	235.0	198.3	158.3
Standard deviation	5.0	2.1	2.5	5.0	2.9	2.9
Pore volume (cm^3^/g)	0.355	0.299	0.220	0.363	0.307	0.245

**Table 6:** Porosity of the M-S-H pastes according to MIP, kerdane and helium pycnometry, autoradiography, and water porosity.

		Mechanically Mixed			Manually Mixed		
	M/S	0.78	1	1.3	0.78	1	1.3
	w/b	1.1	1	1.1	1.45	1.45	1.45
	Age (days)	120	430	120	430	120	430
MIP	Porosity (%)	46.54	39.71	39.17	46.83	46.38	-
Kerdane and helium pycnometry	Bulk density (g/cm^3^)	0.83	0.88	0.84	0.75	0.67	0.71
	True density (g/cm^3^)	2.23	2.30	2.22	2.30	2.34	2.31
	Porosity (%)	62.86	61.78	61.89	67.60	71.39	69.17
Auto-radiography	Porosity (%)-Aged of 180 days	-	-	-	75.2	-	66.4
Water Porosity	Porosity (%)	77.62	70.08	71.03	86.14	78.79	74.69

## Appendix A

**Table A1:** Polishing protocol of sections for MEB/EDS.

Step	Abrasive Paper	Retention Strength	Speed (rd/min)	Time (min)	Abrasive Product	Lubricant + Rinsing Agent
1	240	30 N	500	1–3		Ethanol
2	1200	30 N	250	1		Ethanol
3	2000	30 N	250	1		Ethanol
4	4000	30 N	250	1		Ethanol
5	3 μm	Manual	250	5	Diamond powder (3 μm)	Ethanol
6	1 μm	Manual	250	5	Diamond powder (1 μm)	Ethanol
